# Diagnostic tests to mitigate the antimicrobial resistance pandemic—Still the problem child

**DOI:** 10.1371/journal.pgph.0000710

**Published:** 2022-06-30

**Authors:** Cecilia Ferreyra, Birgitta Gleeson, Otridah Kapona, Marc Mendelson

**Affiliations:** 1 FIND, Geneva, Switzerland; 2 Zambia National Public Health Institute, Lusaka, Zambia; 3 Division of Infectious Diseases and HIV Medicine, Department of Medicine, Groote Schuur Hospital, University of Cape Town, Cape Town, South Africa; PLOS: Public Library of Science, UNITED STATES

The UK Government’s 2015 Antimicrobial Resistance (AMR) Review [1] recommended that no antimicrobial should be prescribed without performing a rapid diagnostic test to prove its need [2]. Seven years on, this remains purely aspirational, and diagnostics continue to be the problem child of the AMR pandemic. COVID-19 has demonstrated just how far we still are from such a goal; despite rapid diagnostics for COVID-19 being developed within months of identification of SARS-CoV-2, the virus was able to drive global, large-scale inappropriate antibiotic prescribing for unsubstantiated bacterial coinfection, just as the common cold and other respiratory viral infections have done for decades. Indeed, despite a pooled prevalence of bacterial or fungal coinfection in patients admitted to hospital with COVID-19 in single figures [3], between 61–100% of those patients received an antibiotic [3–5]. Tellingly, during the first wave of the pandemic in the UK, only 18% of hospitalized patients underwent a diagnostic test to confirm bacterial infection [6]. A lack of understanding of the value of diagnostics tests in directing appropriate antimicrobial use, coupled with fear of COVID-19 infection, also directly increased over-the-counter use of antimicrobials in low- and middle-income countries (LMICs) where antimicrobial control and access to healthcare is limited [7, 8].

Lack of diagnostics, rapid or otherwise, also continues to challenge our understanding of the true global burden of AMR. A recent report from the Global Research on Antimicrobial Resistance (GRAM) project in *The Lancet* estimated that 4.95 million deaths were associated globally with bacterial AMR in 2019. Of these, 1.27 million deaths–more than HIV/AIDS and malaria combined–were directly attributable to resistance [9]. This estimate, based on statistical modelling of over 470 million pieces of global data, has been hailed as the most robust and comprehensive estimate of the global burden of AMR to date, but is likely just the tip of the iceberg. As acknowledged by the GRAM report’s authors, AMR surveillance data from many LMICs are extremely limited; although the statistical model attempted to account for this, it is probable that the burden of death from AMR in LMICs was vastly underestimated, similar to the underestimation of COVID-19 deaths. Furthermore, AMR deaths are projected to increase over time [10], and the ever-present challenge of delivering a health service without effective antimicrobials speaks to the enormous morbidity that AMR is capable of reaping, along with high mortality.

To date, investment in diagnostics to aid in mitigating AMR has largely focused on increasing laboratory surveillance capacity. While AMR surveillance data from LMICs remains limited, the Fleming Fund and other investors in laboratory capacity have helped to improve data availability [11]. The effect of this on appropriate prescribing at the front line, however, remains undetermined. In regions where most prescribing is empiric, such as South East Asia and Sub-Saharan Africa, there remains an urgent need for point-of-care rapid diagnostics if we are to have an impact on misuse of antimicrobials in human health [12]. A case in point is the appropriate use of antimicrobials for children presenting with undifferentiated fever in low-resource settings where bacterial, viral, and malarial infections predominate. C-reactive protein (CRP), the most commonly used diagnostic test to differentiate bacterial from viral infections, is non-specific and may be elevated in a number of non-bacterial infections including malaria and severe dengue fever [13, 14], and with particular relevance to the current global pandemic, COVID-19 [15, 16]. Eight years since the launch of the Longitude Prize, which aims to reward the first innovators to develop a point-of-care diagnostic test that will conserve antimicrobials and is accurate, rapid, affordable and easy to use anywhere in the world, we are still waiting for a winner [17]. Current commercially available diagnostics (Table 1) are not fit-for-purpose in low-resource settings, where diagnostics to drive antimicrobial stewardship is most needed. Although COVID-19 has led to a dramatic increase in capacity for molecular testing in many countries, which can be repurposed to improve access to diagnostics for AMR as well as other common diseases as the pandemic wanes, cost and logistical considerations limit use of such tests at the primary and community healthcare levels.

**Table 1:** Examples of commercially available automated platforms for identification of bloodstream infections, antimicrobial resistance, or susceptibility testing.

Company	Product name	Purpose	Reason for unsuitability to LMICs
Accelerate Dx	Accelerate Pheno	ID & AST	High cost, low throughput
Abacus Diagnostica	GenomEra CDX system	Only MRSA/SA & *Streptococcus pneumoniae*	Limited panel
bioMérieux	BioFire FilmArray	ID & AMR	High cost
Cepheid, a subsidiary of Danaher	GeneXpert	Only MRSA/SA	Limited panel
Curetis	Unyvero System	ID & AMR	High cost, multiple instruments
GenMark Diagnostics	ePlex System	ID & AMR	High cost
GENOMICA S.A.U.	CLART technology	ID & AMR	High complexity
iCubate	iC System	ID & AMR	Limited panel
Luminex/Nanosphere	Verigene	ID & AMR	High cost, separate sample preparation
Master Diagnóstica	Sepsis Flow Chip	ID & AMR	Not integrated, large instrument
OpGen	AdvanDx PNA FISH	ID	ID only, manual workflow, limited panel
Q-linea	ASTar	AST	High cost, AST only
QuantaMatrix	QMAC dRAST	AST	High cost, AST only
Vela Diagnostics	Great Basin Analyser System	*Staphylococcus* species ID & AMR	Limited panel

AMR, antimicrobial resistance (genotypic); AST, antimicrobial susceptibility testing (phenotypic); ID, identification; MRSA, methicillin-resistant *Staphylococcus aureus*; SA, *Staphylococcus aureus*.

Investment in diagnostic development for AMR remains substantially below that for new antimicrobials [18]. Furthermore, access to diagnostic tests at the primary healthcare level in LMICs is poor [19], and tests for infections other than HIV, tuberculosis and malaria must often be paid for by the user, incurring out-of-pocket expenses that are rarely affordable. The Access to COVID-19 Tools (ACT)-Accelerator, a global collaboration to accelerate development, production, and equitable access to COVID-19 tests, treatments and vaccines, was able to reduce the cost of COVID-19 rapid tests to LMICs to less than US$3 (and in some instances less than US$1 [20]), and has provided funding for procurement of at least 128 million diagnostic tests [21]. As the cost of taking global action against AMR will be high (up to $40 billion US dollars per decade as estimated by the O’Neill report [1]), programmes similar to the ACT-Accelerator are likely to be required to support mitigating AMR in LMICs.

Distinguishing between bacterial and viral infections is just one enabler of antimicrobial stewardship. Another is identifying the causative pathogen and understanding its resistance profile. Nucleic acid-based tests have revolutionized the field of diagnostics, enabling clinicians to identify pathogens and their resistance genes in a ‘plug and play’ system within one hour [22]. Next-generation sequencing (NGS), which has proven to be a powerful tool for identification of SARS-CoV-2 mutations that predict ‘resistance’ to vaccines or therapies during the COVID-19 pandemic, could provide equally rapid access to information that would change antibiotic management both at the individual and population level. However, the current prerequisite for a positive bacterial culture prior to NGS is a limiting factor that will require a solution, and the perceived lack of commercial incentives and evidence supporting the market potential for AMR diagnostics in LMICs means that current molecular technologies are not designed to withstand the environmental and funding constraints of low-resource settings. Furthermore, interpretation of resistance mutations continues to be a bottleneck, and advancements will require a concerted effort similar to the cataloguing work performed for *Mycobacterium tuberculosis* mutations [23]. Developments in phenotypic automation and microfluidics are progressing but the potential of these technologies for tackling AMR has not yet been fully achieved in clinical settings. Without progress on these fronts, laborious phenotypic determination of antimicrobial susceptibilities will remain the main work effort of most microbiology laboratories.

AMR may not be as palpable a pandemic as COVID-19, but with an annual death toll in the same order of magnitude, it represents a clear and present danger. The same impetus and enthusiasm for investment and prioritization of diagnostics to help end the COVID-19 pandemic needs to be applied to AMR. Without this, Jim O’Neill’s ambition [2] of using diagnostics to direct all antimicrobial therapy will remain merely an aspiration, and we will still be counting the cost of the lack of diagnostics at the point-of-care and escalating deaths from AMR into the next decade.
